# Analysis of Hormone Regulation on Seed Germination of Coix Based on Muli-Omics Analysis

**DOI:** 10.3390/plants12142700

**Published:** 2023-07-20

**Authors:** Donghao Tuo, Jiawen Wu, Juan Zou, Guoqing Dong, Wanyong Zeng, Jinhua Li, Dengxiang Du

**Affiliations:** School of Life Science and Technology, Wuhan Polytechnic University, Wuhan 430023, China

**Keywords:** *coix lachryrma-jobi* (coix), transcriptome, metabolome, seed germination, hormone-signaling

## Abstract

Seed germination is an important stage of growth and reproduction and plays an important role in the life cycle of spermatophyte. It is co-determined by both genetic and environmental factors, and plant hormone regulation may be a highly conservative mechanism. *Coix lachryrma-jobi* (coix) is a grain with balanced nutrition for medicine and food and has substantial production value. It is an important part of agricultural production, and the efficiency of seed germination after sowing is a key link. In this study, coix species “small white shell Xingren” was used as the experimental material, and changes in gene expression levels and metabolite enrichment in seeds were identified by transcriptome and metabonomic analysis before and after seed germination. A total of 599 metabolites, including those from amino acid metabolism, sugar metabolism, and fatty acid metabolism, were significantly increased in germinating coix. Simultaneously, 10,929 differentially expressed genes (DEGs) were identified, and functional clusters of genes were also significantly clustered in hormone-signaling and glucose and fatty acid metabolism. In addition, this study found that a considerable number of hormone-signaling genes were significantly up-regulated during seed germination, activating multiple metabolic processes. The results of our conjoint analysis of multi omics showed that glucose and fatty acid metabolism played an important role in seed germination under hormone regulation.

## 1. Introduction

Seeds serve a crucial function as the reproductive system and play a vital role in the life cycle of higher plants. Seed dormancy and germination are keys to plant growth and development [1,2]. The seed remains dormant to survive the adverse climate in the unsuitable environment for plant growth; when the environment is suitable for plant growth, seeds start germinating [3,4]. Therefore, seed dormancy and germination are research hotspots in plant molecular biology, and their regulatory mechanisms have been constantly analyzed from different perspectives.

Seed dormancy and germination is a very complex physiological and biochemical process. Environmental factors such as suitable temperature, moisture content, and oxygen content are critical for seed germination [4,5]. Internal cause refers to whether the seed structure is intact, has enough reserves, and can use these reserves to initiate various biochemical reactions related to life activities after receiving external signals [6]. At the molecular level, studies on the regulation of seed dormancy and germination mainly focus on changes in seed-dormancy-related proteins [7], endogenous plant hormone content [8], transcription factors [9], and cis-elements [10]. During seed germination, internal hormone levels change and external environmental factors alter gene transcription and protein levels through complex signal transduction pathways to closely regulate the germination process and prepare for subsequent growth and development. To understand the physiological and biochemical mechanisms of high-vigor seed formation, we can explore the differences and connections at various levels of gene [9,11], transcription, translation, and expression from the perspective of omics [12,13,14].

With the continuous development and improvement of sequencing technology, many studies have reported the omics mechanisms of seed development, germination, and seed vigor [15]. Transcriptome analysis of the mechanism of the wheat germination process showed that seed germination involves complex network regulations, such as substance transport, hormone metabolism, protein degradation, and signal transduction [16]. Some differentially expressed genes and related metabolic pathways were also identified [17]. Li et al. used metabolomics to study rice varieties with different genetic and ecological diversities and detected 121 metabolites; furthermore, 214 differentially expressed metabolites were identified during seed development and growth [18]. The research results of Han et al. showed that during the germination of wheat seeds, there are 82 significantly different metabolites between the embryo and endosperm, involving multiple metabolic pathways [19]. 

*Coix lachryrma-jobi* (coix) has both medicinal and edible value and is a typical medicinal and edible homologous crop [20]. Coix is a nutritious and balanced grain and contains many essential amino acids and trace elements; its protein content is much higher than that of wheat and rice. Most importantly, coix is known as “the king of grass plants” and “pearl of medicine” because of its high medicinal and nutritional value as a medicinal and edible plant [21,22]. Hence, there has been a gradual shift towards recognizing and harnessing the healthcare functions, developmental potential, and practical applications of coix. Seed germination is an important part of production, which not only affects the yield, but also ensures the use of coix to prepare sprouts and other products. Due to its late start, research on seed germination of coix is still relatively basic; research on the germination of coix seeds mainly focuses on exploring the physiological changes during the germination process [23,24], and research on its molecular regulatory mechanism is very limited compared to other cereal crops. Studies on wheat, rice, and other crops have shown that combined transcriptome and metabolome analysis effectively analyze complex characters. This study is of great significance for analyzing the transcriptome and metabolome of germinated and non-germinated coix seeds.

## 2. Results

### 2.1. Transcriptome Analysis during the Seed Germination

An overview of the transcriptome analysis reads is listed in Table 1. Approximately 52,445,384, 62,134,646, 56,839,896, 60,134,670, 56,513,236, and 47,092,678 clean reads pairs were generated, and 50.28 GB of clean data were generated. The correlation coefficient of different materials is 0.608–0.811. The correlation between repetitions of the same material is significantly higher than that between materials, indicating that each replicate could be used for subsequent analysis, which showed a high correlation, and the change in traits may be caused by the difference in the expression changes of certain genes.

**Figure 1:**
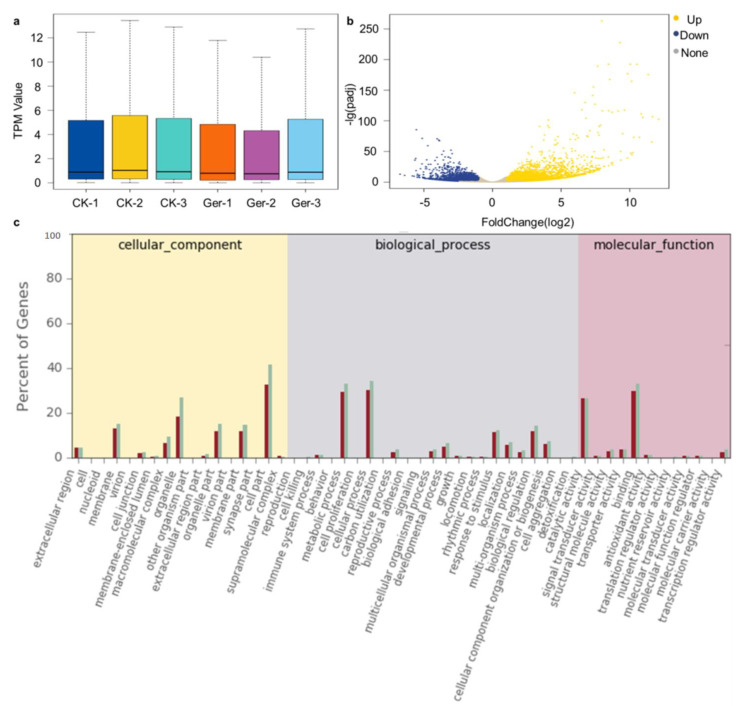
Gene expression analysis during seed germination. (**a**), Expression density of genes using the transcript per million mapped read (TPM) value. The abscissa is the sample name, and the ordinate is the TPM value. The box chart for each region is divided into five statistics (top to bottom are the maximum, upper quartile, median, lower quartile, and minimum). (**b**), Identification of differentially expressed genes (DEGs) between treatments. The volcano plot presents the expression of the DEGs in different treatments, the yellow dots represent up-regulated genes, and the blue dots represent down-regulated genes. (**c**), The GOs classification of all DEGs of germinated seeds and controls. The abscissa is the number of differentially expressed genes in different GOs, and the ordinate is the type of GO enrichment.

A total of 67,257 genes were identified with TPM ≥ 0.1 present in at least one sample; in all samples, low expression genes (about 42%) and medium expression genes (about 30%) occupied the largest proportion. Among the detected genes, 47,170 genes were detected in both periods, 5157 genes were detected only in the non-germination stage, and 14,930 genes were specifically expressed after germination (Figure 1a). There were 10,929 DEGs with significant variations identified between control (CK) and germinated seeds (Ger), among which 6565 genes were up-regulated and 4364 genes were down-regulated (Figure 1b). 

The differentially expressed genes identified at two stages of seed germination were compared with the Gene Ontology (GO) database to obtain detailed annotations of differentially expressed genes (Figure 1c). GO divides gene functions into three categories: biological process, cellular component, and molecular function. Differentially expressed genes were annotated to 53 functional groups, including 13 biological processes, 17 cellular components and 12 molecular functions. Seven GOs, extracellular region, membrane, macromolecular complex, organelle, organelle part, membrane part, and cell part, belong to cellular component, with the most genes enriched. Seven GOs, metabolic process, cellular process, developmental process, response to stimulus, localization, biological regulation, and cellular component organization or biogenesis, belong to biological process, with the most genes enriched. Catalytic activity and binding were two GOs with the most genes enriched in molecular function.

**Figure 2:**
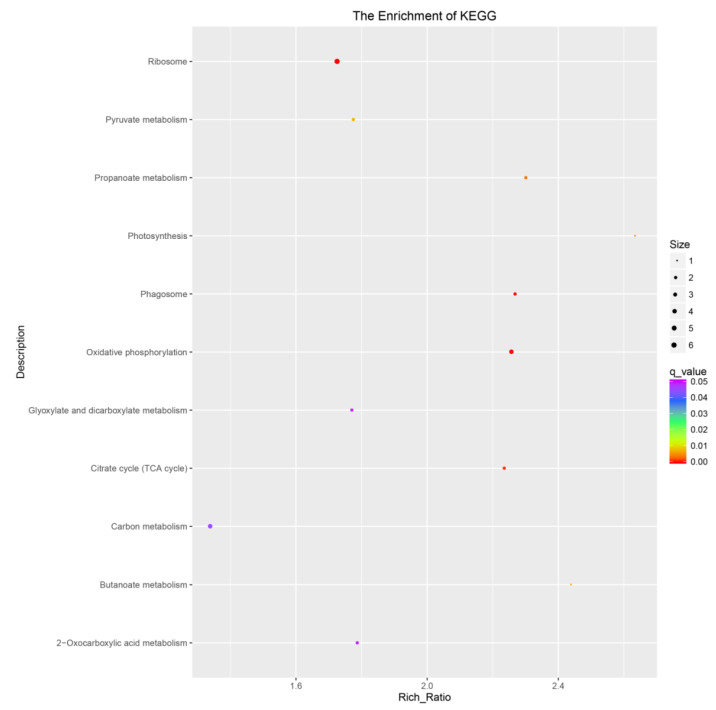
The KEGG pathways of all DEGs of germinated seeds and controls. The abscissa is the percent of differentially expressed genes in different KEGG, and the ordinate is the type of KEGG enrichment.

When subjected to a Kyoto Encyclopedia of Genes and Genomes (KEGG) pathway analysis, using Q-value less than 0.05 as the significant enrichment threshold for all the DEGs for KEGG pathway, the significantly enriched 11 pathways are shown in Figure 2. All the pathways were divided into cellular processes, environmental information processing, genetic information processing, metabolism, and organismic systems. The differentially expressed genes were mainly enriched in pathways such as carbon metabolism, glyoxylate and dicarboxylate metabolism, 2-oxocartboxylic acid metabolism, ribosome, oxidative phosphorylation, and citrate cycle (TCA cycle).

### 2.2. Metabolic Profiling during the Seed Germination

**Figure 3:**
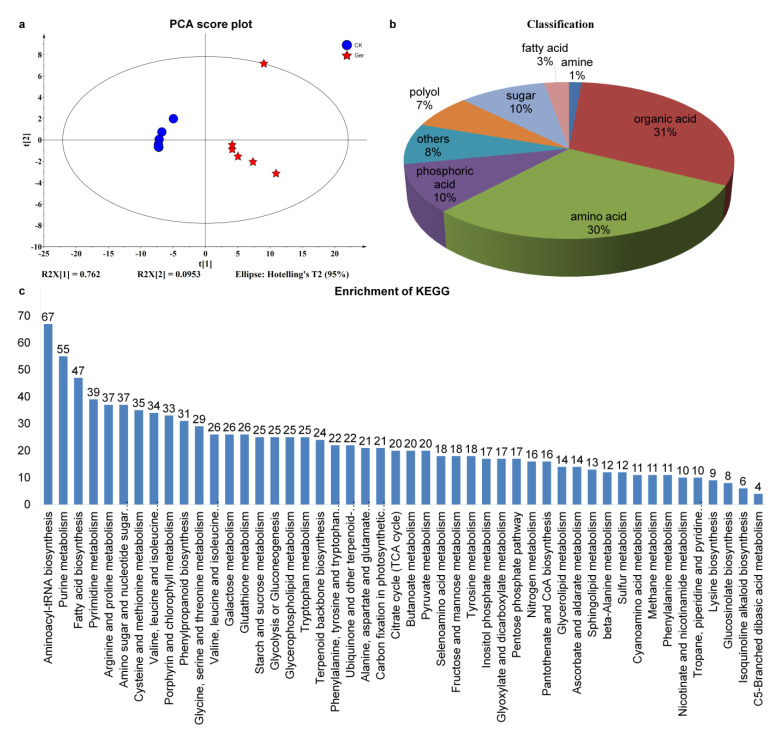
Statistical description of metabolic analysis of coix seed germination. (**a**), Principal Component Score (PCA) analysis of metabolome sequencing results. The X-axis represents the first principal component (PC1), and the Y-axis represents the second principal component (PC2). (**b**), Statistical description of metabolic analysis of coix seed germination. (**c**), The KEGG pathways of all DEGs of germinated seeds and controls. The abscissa is the type of KEGG enrichment, and the ordinate is the percent of differentially expressed genes in different KEGG.

In this study, untargeted metabolomics was used to detect the metabolic components of seeds incubated for 96 h, compared with the non-germinated seeds as control. Principal component analysis (PCA) of metabolomics data showed that R2X [1] and R2X [2] were 0.762 and 0.0953, respectively (Figure 3a). The metabolic components of coix seed groups were relatively scattered before and after germination, indicating that there were great differences in metabolic components of coix seed groups that began to germinate compared with non-germinated seeds. A total of 599 metabolites were detected; the annotated metabolites were classified according to their chemical structures, as shown in Figure 3b. Among them, 31% belong to organic acid, 30% to amino acid, 10% to sugar, 7% to polyol, 10% to phosphoric acid, 3% to fatty acid, 1% amine, and 8% others.

Through the screening of metabolites, 49 metabolites with different changes were shown in Figure 3c, among which 48 were significantly up-regulated and enriched after seed germination, except for the significantly down-regulated and enriched urea content. According to KEGG, metabolites were divided into 48 categories, mainly including aminoacyl–tRNA biosynthesis, purine metabolism, fatty acid biosynthesis, pyrimidine metabolism, arginine and proline metabolism, amino sugar and nucleotide sugar metabolism, cysteine and methionine metabolism, valine, leucine and isoleucine degradation, porphyrin and chlorophyll metabolism, phenylpropanoid biosynthesis, glycine, serine and threonine metabolism, valine, leucine and isoleucine biosynthesis, galactose metabolism, glutathione metabolism, starch and sucrose metabolism, glycolysis or gluconeogenesis, glycerophospholipid metabolism, tryptophan metabolism, terpenoid backbone biosynthesis and phenylalanine, and tyrosine and tryptophan biosynthesis. The enriched differential metabolites were significantly correlated with the metabolites detected in some existing seed germination studies, and the significant enrichment of 0.059341 (Raw P) was also detected in the aminobutyric acid metabolism process concerned in this study.

### 2.3. Correlations between Transcriptome and Metabolites

**Figure 4:**
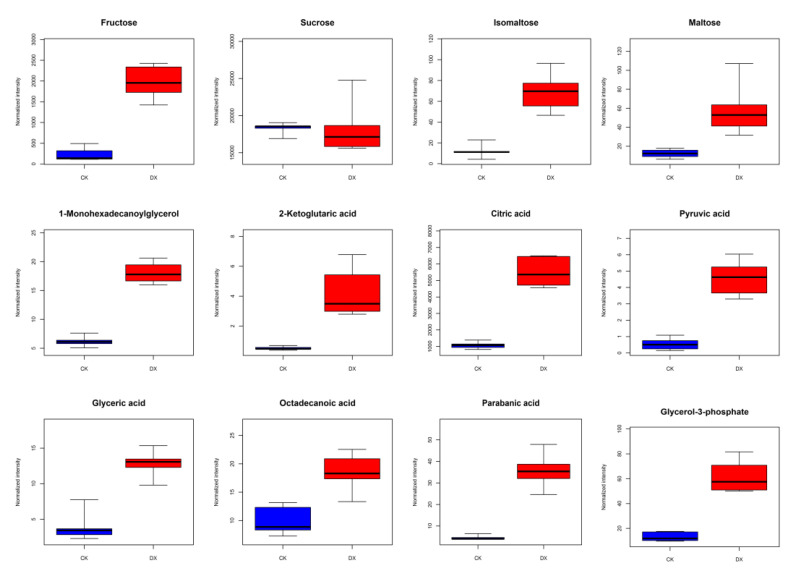
Differential metabolites and differentially expressed genes related to GABA synthesis pathway. In the box plot, the abscissa represents the sample and the ordinate represents the enrichment content for the differentially enriched metabolites. Blue is the test result of non-germinated seeds, and red is the test result of germinated seeds.

Comprehensive analysis of transcriptomic and metabolomic data showed that amino acid metabolism, carbohydrate metabolism, and lipid metabolism significantly changed during seed germination, thus initiating the biological processes of coix. Part of the metabolite change is shown in Figure 4, fructose, sucrose, isomaltose, and maltose, which were the most significant increases of water-soluble sugar in the seeds, and also shown in the growth of the genes that were associated with the metabolic metabolism. Metabolites associated with fatty acid metabolism were detected, for example 1-Monohexadecanoylglycerol, 2-Ketoglutaric acidα, citric acid, pyruvic acid, glyceric acid, octadecanoic acid, parabanic acid, and glycerol-3-phosphate were significantly up-regulated content detected during germination in seeds. 

**Figure 5:**
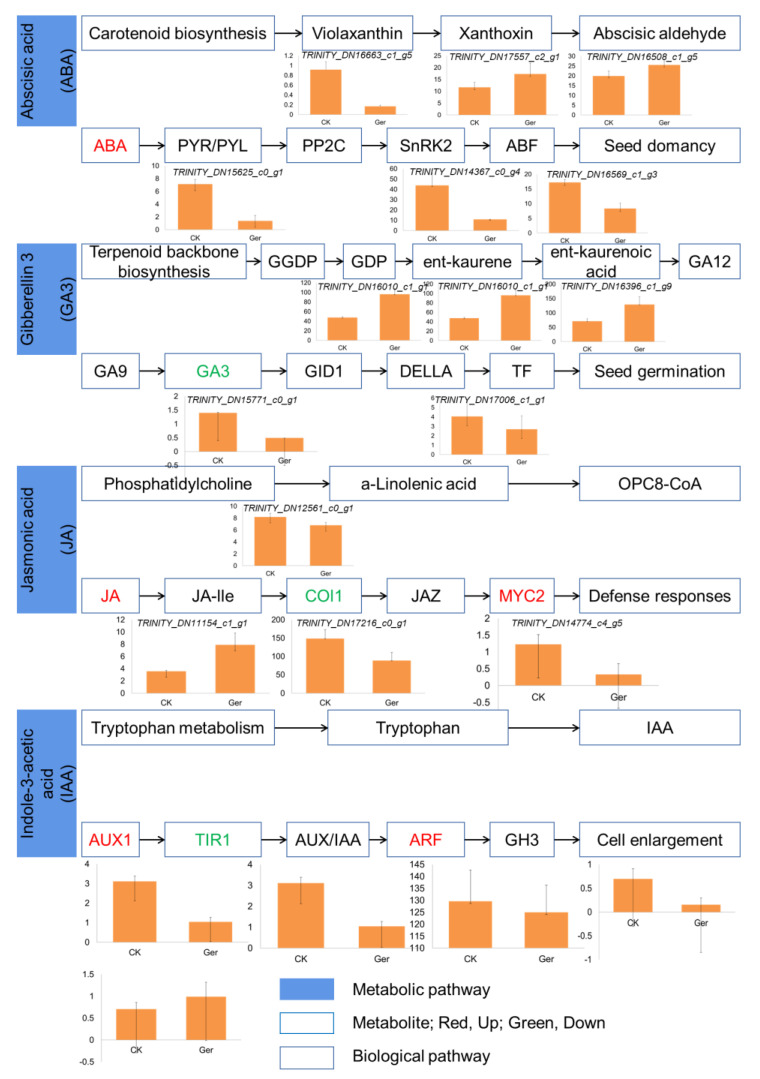
Hormone regulatory pathways change during seed germination. Analysis of metabolite accumulation in hormone metabolism pathway shows that red indicates up-regulation and enrichment, and green indicates down-regulation and enrichment. The column chart shows some differentially expressed genes in the pathway, with the gene name in italic font.

Seed germination is mainly regulated by the synergism of various endogenous plant hormones. During the germination of coix seeds, 495 genes were expressed, including 254 differentially expressed genes. In general, DEGs involved in plant hormone signal transduction were significantly different, including ABA signal, GA signal, JA signal, IAA signal, cytokinin signal, and brassinosteroid signal, observed at the early stages of seedling growth. In order to study the relationship between plant hormone pathways and seed germination, as shown in Figure 5, each hormone signaling pathway has corresponding transcription factors involved and is regulated by related enzymes and metabolites.

The expression of ABA synthesis and signal transduction enzymes in dried seeds is high, but the expression decreases significantly after immersion in water, such as the synthesis of the key enzyme NCEB, the plant non-fermenting 1 related protein kinase (SnRK2), and the transcription factor ABF in signal transduction. As shown in Figure 5, four genes were down-regulated after germination, while two genes were up-regulated. Gene *TRINITY_DN15625_c0_g1* is homologous to the PYR/PYL gene in Arabidopsis, and gene *TRINITY_DN14367_c0_g4* and *TRINITY_DN16569_c1_g3* belong to SnRK2 and ABF, respectively. ABA is consistent with the decrease in the expression of related genes such as LEA and HSP in seeds, which may control seed dormancy by regulating genes related to late seed development.

In contrast, the synthetases in the GA pathway are ent kaurene oxidase and ent kaurene oxidase. The expression of important signal molecules GA insensitive dwarf genes (GA insensitive genes *TRINITY_DN16010_c1_g1*, *TRINITY_DN16010_c1_g1*, and *TRINITY_DN16396_c1_g9*) and transcription factor GAMYB increased significantly after water absorption. GAMYB can promote the expression of related genes by binding to the cis element GARE of downstream genes, thereby activating metabolism. Among other plant hormones, the synthesis of lyric acid (JA) and the expression of genes related to signaling pathways have an overall trend of increasing, which is inconsistent with the reported negative regulatory effect of JA on germination. In addition to J, other plant hormone synthesis and signal-related gene expression patterns are not consistent, so it is not possible to clarify their expression changes.

## 3. Methods

### 3.1. Plant Materials

Healthy coix seeds of the same size were selected, disinfected with 1% sodium hypochlorite, and then placed in a petri dish (9 cm in diameter) covered with two layers of filter paper, with 30 seeds in each dish. The seeds treated above were placed in an incubator at 25 °C for cultivation. After 96 h, the germinated seeds were sampled for transcriptome and metabonomics analysis, and treated seeds were used as controls.

### 3.2. High Throughput Transcriptome Analysis 

Total RNA from seeds was extracted using a total RNA extraction kit and sequenced using the Illumina HiSeq platform [25,26]. HISAT2 V2.1.0 was selected for hierarchical indexing for spliced alignment of transcripts to compare transcriptome sequencing reads to the reference genome [27]. The transcript per million mapped read (TPM) value of each gene was calculated using cuff links, and the add counts of each gene were obtained using htseq-count [28]. DEGs were identified using the DESeq package function to estimate size factors and nbinomTest. A *p*-value < 0.05 and fold change > 2 or fold change < 0.5 were set as the threshold for significantly differential expression. Gene Ontology (GO) enrichment and Kyoto Encyclopedia of Genes and Genomes (KEGG) pathway enrichment analyses of DEGs were performed using R based on the hypergeometric distribution of data from http://www.geneontology.org/ (accessed on 16 March 2023) and http://www.genome.jp/kegg/ (accessed on 16 March 2023), respectively. 

### 3.3. Sequencing and Bioinformatics Analysis of Untargeted Metabolomics

Untargeted metabolomics was used to detect metabolites of coix seeds before and after germination. Non-targeted metabolic profiling analyses were performed using a Perkin Elmer 680 GC (Perkin Elmer Inc., Akron, OH, USA) and Q Exactive Focus Orbitrap LC-MS/MS (Thermo Scientific, Waltham, MA, USA). The recorded data were processed with compound discoverer (CD) 3.1 software to obtain the mass-to-charge ratio, retention time, and MS/MS2 information of all detected substances. The detected signals were automatically matched through the internally established reference libraries of chemical standard entries of software to predict and identify metabolite information. Multiple reaction monitoring (MRM) mode with QTRAP 6500+ LC-MS/MS (Shimadzu, Kyoto, Japan) was used for targeted metabolome analysis. The detection window was set to 80 s, and the targeted scanning time was 1.5 s. The original data were processed using Multi Quant 3.0.3 software. The chromatographic column was a C18 column (Shim-pack GLSS C18, 1.9UM, 2.1*100, Shimadzu). 

The total peak area was normalized by dividing each metabolite in the sample by the total peak area of the sample to normalize the data before analysis. Principal Component Score (PCA) analysis was performed using the R software package ropls to classify and discriminate between samples. The differences in accumulated metabolites (DAMs) between different control groups were determined based on screening criteria FC > 1.50, *p* value < 0.05, and VIP > 1 [29]. Kyoto Encyclopedia of Genes and Genomes (KEGG) enrichment analysis was performed on the DAM using the KOBAS software [30].

## 4. Discussion

Seeds are essential for all seed plants as they enable the process of sexual reproduction necessary for species perpetuation. Seed germination determines the success of plant reproduction and has substantial economic value. Seeds undergo a complex process from maturity to germination and subsequent seedling establishment. Many factors affect seed germination, including the regulation of physiological and biochemical indicators, genes, and proteins [31,32]. This study analyzed changes in gene expression and metabolites in coix seed at 96 h by high-throughput transcriptome and metabolome analysis. There were 10,929 DEGs and 599 DAMs with significant variations identified between control and germinated seeds. The variation trends of gene expression number and expression amount indicated that a large number of genes were initiated in the early seed germination, and with the increase of gene expression level, various metabolic activities of seeds were driven, and seeds of coix entered a state of enhanced life activities. The differences between DEGs and DAMs indicate that seed germination is a process of enhanced biological activity [33,34]. These phenomena have also been found and elaborated in related studies, demonstrating that physicochemical changes and expression regulation of seed germination are similar in different species [35,36,37]. 

Plant hormones are also crucial factors affecting seed germination, and numerous studies have been conducted on different species [38,39,40]. Xu et al. found that dozens of up-regulated genes were related to plant hormone biosynthesis and signal transduction, including the auxin signaling pathway [41], brassinosteroid biosynthesis, and signal transduction [42], as well as some GA and ABA signal transduction genes, in the pairing comparison between peanut seeds at the early germination stage and post-ripening stage [43,44]. They suggested that the synergistic action of multiple hormone signal transduction networks played a crucial role in radicle protuberance and seed germination. ABA regulates the transition from dormancy to germination and from germination to growth [45]. Endogenous ABA content is the key to determining whether the embryo enters a dormancy state or deactivates dormancy (spike germination) before maturation. Endogenous ABA accumulates in seeds during seed maturation and initiates and maintains seed dormancy, thereby preventing seed germination. In contrast, endogenous ABA levels decrease, and GA content increases before seed germination. Thus, it was concluded that ABA is a critical hormone for maintaining seed dormancy, and GA can counteract the effects of ABA and promote seed germination [46,47]. Wang et al. found that low temperatures (15 °C) inhibited GA signal transduction in rice seeds (*Oryza sativa*), resulting in increased ABA synthesis and delayed rice seed germination [48]. They also found that low-temperature treatment promotes the transport of soluble sugars from the endosperm to the embryo.

Different plant-hormone-pathway-related enzymes and transcription factors exhibit different expression trends in our study. For example, the expression of signal molecules that promote ABA synthesis declined after immersion, while the overall expression of GA- and BR-related genes declined. We detected four down-regulated genes and two up-regulated genes in the ABA pathway, three up-regulated genes and two down-regulated genes in the GA3 pathway, three down-regulated genes and one up-regulated gene in the JA pathway, and four down-regulated genes and one up-regulated gene in the IAA pathway. Rich gene expression differences lead to differences in metabolites. The rich changes in hormone-response-signal-related genes have enriched our understanding of coix seed germination and provided a foundation for further research.

## 5. Conclusions

*Coix* is a crop with edible and medicinal value. Analysis of the germination process enables understanding and application of coix materials. We studied changes in the seed germination process by combining transcriptome sequencing and non-targeted metabonomics with the cultivated coix. Transcriptome and metabolomic data were integrated and evaluated, and various enrichment pathways were identified, including glucose metabolism, amino acid biosynthesis, lipid metabolism, and hormone response pathways. We focused on lipid metabolism and hormone responses and identified some up-regulated or down-regulated genes. The results showed that the regulatory mechanism of coix seed germination might not be a single gene or metabolite but rather a complex regulatory and signaling mechanism. Nonetheless, the precise mechanism through which these candidate genes or metabolites participate in coix seed germination requires further investigation.

## Figures and Tables

**Table 1:** Statistical results of the RNA-Seq reads.

Sample	Raw Reads	Clean Reads	Clean Bases	Error (%)	Q20 (%)	Q30 (%)	GC (%)
CK-1	52,445,384	52,445,384	7.87 G	0.02	98.1	94.7	56.23
CK-2	62,134,646	62,134,646	9.32 G	0.02	97.95	94.37	57.88
CK-3	56,839,896	56,839,896	8.53 G	0.02	98.02	94.56	57.99
Ger-1	60,134,670	60,134,670	9.02 G	0.02	98.13	94.74	56.99
Ger-2	56,513,236	56,513,236	8.48 G	0.02	98.01	94.51	56.3
Ger-3	47,092,678	47,092,678	7.06 G	0.02	97.99	94.47	55.66

Sample: Name of a Sample; Raw reads: Statistics of Raw sequence data, counting the number of sequenced sequences per file in a unit of four acts; Clean reads: the calculation method is the same as Raw reads and Raw bases, except that the statistical files are filtered sequencing data. Subsequent bioinformatics analyses were based on Clean reads; Clean bases: The number of sequenced sequences multiplied by the length of the sequence and converted to G; Error: base Error rate; Q 20, Q 30: percentage of bases with Phred values greater than 20 and 30 in the total number of bases; GC Content: The percentage of the sum of bases G and C to the total number of bases.

## Data Availability

The data of this study has not been publicly disclosed. If necessary, please contact the corresponding author.
